# Self-Assembled Lipoplexes of Short Interfering RNA (siRNA) Using Spermine-Based Fatty Acid Amide Guanidines: Effect on Gene Silencing Efficiency

**DOI:** 10.3390/pharmaceutics3030406

**Published:** 2011-07-13

**Authors:** Abdelkader A. Metwally, Ian S. Blagbrough

**Affiliations:** Department of Pharmacy and Pharmacology, University of Bath, Bath BA2 7AY, UK

**Keywords:** fatty acids, gene silencing, GFP, guanidine, lipoplexes, nanoparticles, self-assembly, siRNA, spermine

## Abstract

Four guanidine derivatives of *N*^4^, *N*^9^-diacylated spermine have been designed, synthesized, and characterized. These guanidine-containing cationic lipids bound siRNA and formed nanoparticles. Two cationic lipids with C18 unsaturated chains, *N^1^*,*N^12^*-diamidino-*N*^4^, *N*^9^-dioleoylspermine and *N^1^*,*N^12^*-diamidino-*N*^4^-linoleoyl*-N*^9^-oleoylspermine, were more efficient in terms of GFP expression reduction compared to the other cationic lipids with shorter C12 (12:0) and very long C22 (22:1) chains. *N^1^*,*N^12^*-Diamidino-*N*^4^-linoleoyl*-N*^9^-oleoylspermine siRNA lipoplexes resulted in GFP reduction (26%) in the presence of serum, and cell viability (64%). These data are comparable to those obtained with TransIT TKO. Thus, cationic lipid guanidines based on *N*^4^, *N*^9^-diacylated spermines are good candidates for non-viral delivery of siRNA to HeLa cells using self-assembled lipoplexes.

## 1. Introduction

Short interfering RNA (siRNA) is a synthetic double-stranded (ds) RNA of 21-25 nucleotides per strand. Post-transcriptional gene silencing by siRNA is an important biological tool in functional genomic studies. Sequence specific gene silencing by siRNA has many potential therapeutic applications [1]. In 1998, Fire, Mello and co-workers reported that genes can be silenced at the post-transcriptional level by short ds RNA segments [2], a discovery that was awarded the 2006 Nobel Prize in medicine or physiology. Elbashir *et al.* proved in 2001 that gene silencing using siRNA is found in mammalian systems [3]. The optimum length of siRNA to affect post-transcriptional gene silencing in mammalian cells is typically less than 30 bp, as it avoids inducing non-specific mRNA degradation due to immune response (interferon) while maintaining sequence specific mRNA degradation [3]. The effector complex for mRNA degradation, the RNA induced silencing complex (RISC), is a complex of proteins and the siRNA with the complementary sequence to the target mRNA. The key protein in the degradation process is the Ago2 protein, one of the argonaute family of proteins, which contains a domain with RNase H (endonuclease) type activity. RISC assembly and function occur in the cytoplasm of the cell [4,5].

**Figure 1.**
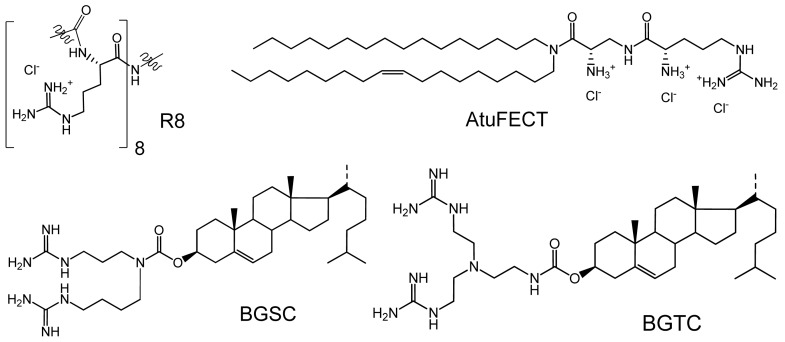
Some guanidines used in delivery of genes and other cargoes.

In order to achieve gene silencing mediated by siRNA, the siRNA should be delivered intact to the cytoplasm of the cell. Due to the negative charge of the siRNA phosphate backbone, and its susceptibility to degradation by various nucleases, a vector is needed to achieve efficient intracellular delivery of siRNA. Cationic lipids are currently under investigation for the non-viral delivery of lipoplexes of DNA and siRNA [6-8]. The polar (cationic) head-group can be an amine (primary, secondary, tertiary, and even quaternary e.g., imidazolium [9]) or guanidine functional group. Guanidines, the most basic functional group in biological chemistry, are positively charged at physiological pH 7.4 as they have p*K_a_* = 12.5 [10]. Guanidines have the extra advantage, being bidentate, of being able to form two hydrogen bonds with negatively charged groups e.g., carboxylates, phosphates or sulfates present on the carbohydrates associated with the cell membrane, and this advantage has been used in vectors e.g., R8, Arg_8_ [11,12] to transport cargoes across cell membranes. These characteristics led to the design of many non-viral vectors for DNA and siRNA, varying from cationic lipids incorporating guanidine head-groups [13-15] e.g., AtuFECT [15], to cationic polymers [16,17] and dendrimers [18,19], to carbohydrate derivatives [19,20], and hydrogels of guanidinylated hyaluronic acid [21]. The use of guanidinium-containing lipid based carriers for gene delivery dates back to 1996 where Lehn *et al.* synthesized two guanidinium cholesterol lipids: bis-guanidiniumspermidine-cholesterol (BGSC) and bis-guanidinium-trencholesterol (BGTC), each containing two guanidine groups, which were synthesized and evaluated for their DNA transfection efficiencies in eukaryotic cells (Figure 1) [22] where they were found to be efficient DNA transfecting agents. Furthermore, BGTC was found to mediate transfection in an aqueous solution without the need to prepare it first in a liposomal form.

In this work, spermine, a naturally occurring polyamine, was acylated with different fatty acids on its secondary amine groups and then guanidinylated at the terminal primary amine groups. The guanidinylated non-viral vectors were characterized and evaluated for their ability to deliver siRNA that targets green fluorescent protein (GFP) in HeLa cells that stably express GFP.

### 2. Experimental Section

#### 2.1. Materials and general methods

Dicyclohexylcarbodiimide (DCC), 1,3-di-Boc-2-(trifluoromethylsulfonyl)guanidine, 4-dimethylamino-pyridine (DMAP), fatty acids, G418, hydrazine monohydrate, *N*-carbethoxyphthalimide, spermine, triethylamine (TEA), and trifluoroacetic acid (TFA) were purchased from Sigma-Aldrich (Gillingham, UK). All solvents were purchased from Fisher Scientific UK (Loughborough, UK). Cell culture media were purchased from Gibco (Invitrogen Ltd, Paisley, UK). HeLa cells stably expressing GFP were obtained from the Cell Service at Cancer Research UK (CRUK, London Research Institute, Clare Hall Laboratories, South Mimms, London, UK). NMR spectra were recorded in deuterio-chloroform using a Bruker Avance III spectrometer operating at 400.13 MHz spectrometer for ^1^H. The high resolution (HR) time-of-flight mass spectra were obtained on a Bruker Daltonics micrOTOF mass spectrometer using electrospray ionisation (ESI). AllStars siRNA negative controls with/without an Alexa Fluor® 647 tag at the 3′-position were purchased from Qiagen (Crawley, UK) as was siRNA against GFP labelled with Alexa Fluor® 647 at the 3′-position of the sense strand, sequences:

$$\begin{array}{c} \text{Sense strand}:5'‐\text{GCAAGCUGACCCUGAAGUUCAUTT}‐3',\\ \text{Anti-sense strand}:5'‐\text{AUGAACUUCAGGGUCAGCUUGCCG}‐3',\\ \text{Target DNA sequence}:5'‐\text{CGGCAAGCTGACCCTGAAGTTCAT}‐3'. \end{array}$$

### 2.2. Synthesis of N^1^,N^12^-diamidino-N^4^,N^9^-diacylated spermines

*N*-Carbethoxyphthalimide (0.44 g, 2 mmol) was added to a solution of 1,12-diamino-4,9-diazododecane (spermine) (0.20 g, 1 mmol) in DCM (10 mL). The solution was stirred 20 °C for 3 h then evaporated to dryness in vacuo and the residue was used directly in the following step. To a solution of 1,12-diphthalimido-4,9-diazadodecane in DCM (10 mL) and TEA (0.28 mL, 2 mmol) fatty acid chloride (2 mmol), or alternatively fatty acid (2 mmol), DMAP (0.24 g, 2 mmol), and DCC (0.4 g, 2 mmol) were added and stirred for 18 h under nitrogen atmosphere. To prepare, 1,12-diphthalimido-*N*^4^-linoleoyl*-N*^9^-oleoylspermine, first, 1,12-diphthalimido-*N*^4^-oleoylspermine was prepared by reacting 1,12-diphthalimido-4,9-diazadodecane (1 mmol) with 1 (mmol) oleic acid using DCC as previously described [23]. After purifying the product over silica gel (DCM/MeOH 20:1 v/v then 10:1 v/v), it was further conjugated to linoleic acid (1 mmol) using DCC as the coupling agent. The solvent was then evaporated (for all of the prepared compounds) to dryness in vacuo and the residue was treated with hydrazine monohydrate (2 mL) in a mixture of DCM (15 mL) and THF (15 mL) and heated under reflux for 4 h. The solvent was then evaporated in vacuo to dryness and the residue purified over silica gel (DCM/MeOH 10:1 v/v then DCM/MeOH/NH_4_OH 20:10:1 v/v/v) to afford the *N^4^*,*N^9^* fatty acid amides of spermine. HRMS of *N*^4^,*N*^9^-dierucoylspermine, *N*^4^,*N*^9^-dilauroylspermine, and *N*^4^,*N*^9^-dioleoylspermine were found as previously described [23]. *N*^4^-Linoleoyl-*N*^9^-oleoyl-1,12-diamino-4,9-diazadodecane HRMS m/z found (M+H)^+^ 729.6980, C_46_H_89_N_4_O_2_ requires (M+H)^+^ 729.6986.

The *N^1^*,*N^12^*-diamidino-*N^4^*,*N^9^*-diacylated spermines were prepared by reacting each of the prepared *N^4^*,*N^9^*-diacylated spermine (1 mmol) with 1,3-di-Boc-2-(trifluoromethylsulfonyl)guanidine (2 mmol) and TEA (2 mmol) in DCM (10 mL) at 20 °C for 24 h. The reaction mixture was then evaporated to dryness in vacuo and the residue was purified over silica gel (DCM/MeOH 100:1 v/v then 100:2 v/v) and the required fractions were concentrated. The residue was then added to DCM (6 mL), TFA (2 mL) was added, and the mixture stirred at 20 °C for 4 h. The reaction mixture was then evaporated to dryness in vacuo to afford the title compounds. *N^1^*,*N^12^*-Diamidino*-N*^4^, *N*^9^-dierucoylspermine **1**, HRMS m/z, ESI found (M+H)^+^ 927.8795, C_56_H_111_N_8_O_2_ requires (M+H)^+^ 927.8825. *N^1^*,*N^12^*-Diamidino-*N*^4^, *N*^9^-dilauroylspermine **2**, HRMS m/z, ESI found (M+H)^+^ 651.5996, C_36_H_75_N_8_O_2_ requires (M+H)+ 651.6008. *N^1^*,*N^12^*-Diamidino-*N*^4^, *N*^9^-dioleoylspermine **3**, HRMS m/z, ESI found (M+H)^+^ 815.7549, C_48_H_95_N_8_O_2_ requires (M+H)^+^ 815.7573. *N^1^*,*N^12^*-Diamidino-*N*^4^-linoleoyl*-N*^9^-oleoylspermine **4**, HRMS m/z, ESI found (M+H)^+^ 813.7384, C_48_H_93_N_8_O_2_ requires (M+H)^+^ 813.7416.

### 2.3. Transfection studies of HeLa cells stably expressing GFP

Cells were trypsinized at confluency 80–90%, seeded at a density of 65,000 cells/well in 24-well plates and incubated for 24 h at 37 °C, 5% CO_2_, prior to transfection. The lipoplexes were prepared by mixing the specified amounts of the transfection reagent in OptiMEM serum-free medium (50 μL) with 15 μL of siRNA (1 μM) in OptiMEM serum-free medium. The solutions were mixed for 2–3 s with a vortex mixer. On the day of transfection, the lipoplex solutions were added to wells containing DMEM (10% FCS) to make the final volume in each well 1 mL (*i.e.*, 6,500 cells/100 μL). The plates were then incubated for 48 h at 37 °C, 5% CO_2_. siRNA against GFP used in these experiments has 24 base-pairs, thus, each molecule of siRNA contains 48 negative charges corresponding to 48 negatively charged phosphate groups in the siRNA backbone. The synthesized spermine fatty acid amides each contain two terminal primary amine groups which will be positively charged at physiological pH 7.4, therefore, each vector molecule carries two positive charges. N/P ratio is calculated using the following equation:

$$N/P=\frac{\text{number of moles of cationic lipid}\times 2}{\text{number of moles of siRNA}\times 48}$$

### 2.4. Flow cytometry (FACS)

For analysis of delivery and then reduction of expression of GFP by flow cytometry, cells were trypsinized and resuspended in complete medium without phenol red. Cells were centrifuged (1000 rpm for 5 min), and washed twice by resuspending in PBS containing 0.1% BSA (1 mg/mL bovine serum albumin) and centrifugation (1000 rpm for 5 min). The collected cells was then resuspended in PBS and transferred to a flow cytometer tube (Becton Dickinson, UK). Cells (typically 10,000–20,000 events) were then analyzed using a FACSCanto flow cytometer (Becton Dickinson, UK), equipped with an argon ion laser at 488 nm for excitation, a Long Pass (LP) filter at 502 nm and a detector at 530 nm (range +/−15 nm) for fluorescence emission, helium/neon laser at 633 nm, and detector for the Alexa Fluor 647 at 660 nm (range +/− 10 nm). GFP expression is calculated as:

$$\%\text{GFP}=\frac{\text{GFP fluorescence of transfected cells}}{\text{GFP fluorescence of control cells}}\times 100$$

### 2.5. Confocal microscopy cell imaging

Cells were trypsinized at confluency 80–90% and were seeded at a density of 65,000 cells/well in 24-well plates that have a round-glass cover slip (12 mm in diameter) and were incubated for 24 h prior to transfection which was carried out as described above (section 2.3). After 48 h, the cell culture media in each well were aspirated and the cells washed with PBS (3 × 0.5 mL). The cell membrane was then stained with wheat germ agglutinin (WGA) conjugated to Alexa Fluor® 555. The concentration of WGA-Alexa Fluor® 555 working solution was adjusted to a concentration of 5 μg/mL in Hank's balanced salt solution without phenol red. The cells were incubated for 10 min in the dye working solution at 37 °C, 5% CO_2_ in the dark. The cells were washed with PBS (3 × 0.5 mL) and then fixed with 4% paraformaldehyde in PBS solution for 20 min at 20 °C in the dark. The cover slips were then removed from each well, washed with PBS (2 × 0.5 mL), left to dry briefly in air, and then mounted on glass slides using Mowiol (polyvinyl alcohol) solution as the mounting media and left in the dark at 20 °C (18 h) to allow hardening of the mounting media. The cells were examined using a Carl Zeiss laser scanning microscope LSM 510 meta, with GFP excitation 488 nm, emission 505-550 nm (band pass filter), Alexa Fluor® 555 excitation 543 nm, emission 560-615 nm (band pass filter), and Alexa Fluor® 647 excitation 633 nm, emission 657–753 nm (meta detector).

### 2.6. Cell viability assay

Cells were seeded at a density of 6,500 cells per well of 96-well plates. The transfection was carried out using the same protocol as transfecting the 24-well plates with the exception of reducing the amount of lipoplexes such that each well contains 1.5 pmol siRNA in a final volume of 100 μL/well. After 44 h, alamarBlue® [24] (10 μL) was added to each well. After incubation (3.5 h), the absorbance of each well was measured at 570 nm and 600 nm and calculations were carried out according to the standard protocol provided by the supplier.

### 2.7. Particle size and zeta potential measurements

Lipoplexes were prepared by adding siRNA solution (75 μL, 1 μM) in HEPES (pH 7.4, 10 mM) to HEPES (250 μL) containing the specified amount of transfection reagent followed by vortex mixing for 4 s. Samples were then diluted to a final volume of 3 mL by HEPES buffer. Samples were mixed for 10 s directly before measurements. Measurements were carried out using Malvern Zetasizer Nano S90 using refractive index 1.59, viscosity 0.89 cP, dielectric constant 79, and temperature set to 25 °C with equilibrium time 3 min. Z-Average diameter in nm and zeta potential in mV were recorded as averages of three and six measurements respectively.

### 2.8. siRNA binding (RiboGreen intercalation assay)

RiboGreen (Invitrogen) working solution was prepared by diluting RiboGreen stock solution 1 to 400 in TE buffer (10 mM Tris-HCl, 1 mM EDTA, pH 7.5 diluted 1 to 20 in RNase free water). RiboGreen working solution (40 μL) was added to each well of a 96-well plate (black bottom) containing free siRNA (1 pmol) or complexed with lipospermines in TE buffer at the lipid/siRNA ratios that showed the best reduction in GFP expression. Each well contained a final volume of 120 μL. The fluorescence was measured using FLUOstar Optima Microplate Reader (BMG-LABTECH), λ_ex_ = 480 nm and λ_em_ = 520 nm. The amount of siRNA available to interact with the lipid vector was calculated by subtracting the values of RiboGreen background fluorescence (RiboGreen without siRNA) from those obtained for each measurement, and expressed as a percentage of the control that contained naked siRNA only according to the following equation:

%free siRNA=100×RiboGreen fluorescence of complexes/RiboGreen fluorescence of naked siRNA

## 3. Results and Discussion

### 3.1. Synthesis of N^1^,N^12^-diamidine derivatives of spermine

We have designed a series of novel lipoguanidines based upon our recently published lipopolyamines [23] in order to investigate the SAR of replacing primary amines with guanidine functional groups. These are formally called di-imidamides of alkanes and the nomenclature also permits *N*-aminoiminomethyl. Where we have referred to them as guanidines, they are more correctly *N*-amidines of spermine.

Our four lipoguanidines will be investigated in terms of their efficiency and their effect on cell viability as non-viral vectors for siRNA delivery. An amidine group was attached to each of the two terminal primary amines of spermine to result in the di-guanidines (*N^1^*,*N^12^*-diamidino-amines). Three fatty acids of different chain length and saturation were used to synthesise the lipoguanidines **1**, **2**, and **3** by acylation at *N*^4^ and *N*^9^ of spermine. The fourth lipoguanidine **4** was synthesized by acylating sequentially using two different long-chain fatty acids (linoleic and oleic) to *N*^4^ and *N*^9^.

**Figure 2.**
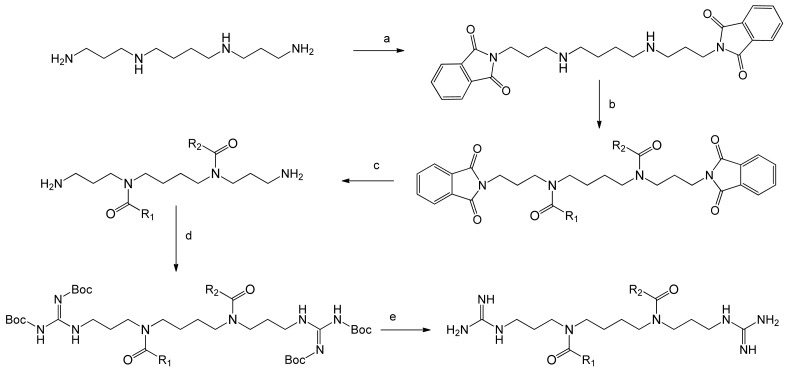
Synthesis of *N^1^*,*N^12^*-diamidino-*N^4^*,*N^9^*-diacylated spermines. a: *N*-Carbethoxyphthalimide, DCM; b: fatty acid, DCC, TEA; c: hydrazine monohydrate, DCM/THF 1:1 mixture; d: 1,3-di-Boc-2-(trifluoromethylsulfonyl)guanidine, TEA, DCM; e: TFA, DCM.

**Figure 3.**
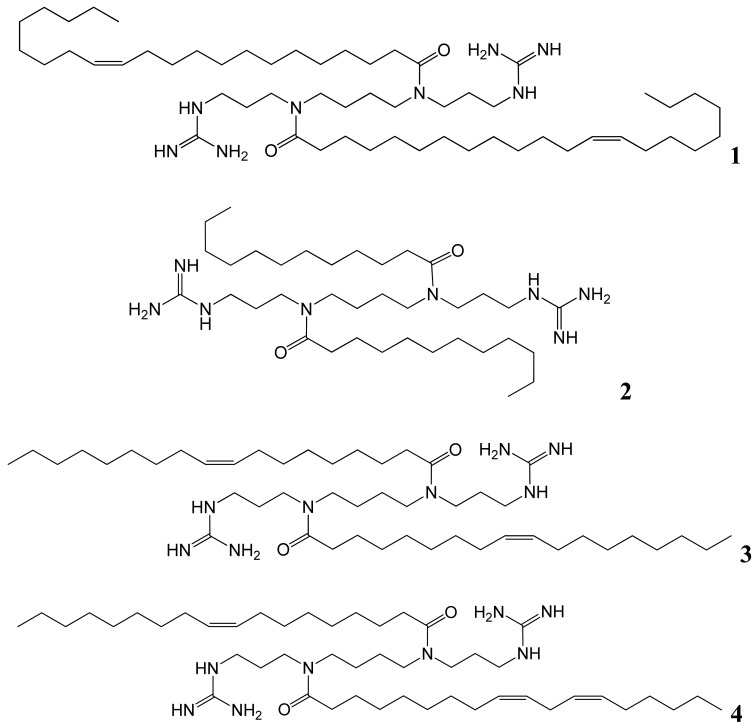
*N*^1^,*N*^12^-Diamidine derivatives of different lipospermines.

The synthesis of the guanidinylated *N^4^*,*N^9^*-diacylated spermine conjugates started with the synthesis of the *N^4^*,*N^9^*-difatty acids spermine derivatives (Figure 2). The symmetrical lipospermines; *i.e.*, those with the same fatty acid chains conjugated to positions *N^4^* and *N^9^* of the spermine chain was carried out as described previously [23]. For the synthesis of the unsymmetrical *N*^4^-linoleoyl*-N*^9^-oleoylspermine, the primary amine groups of spermine were selectively protected with the phthalimide protecting group (2 eq. of *N*-carbethoxyphthalimide in CH_2_Cl_2_). Then one oleoyl chain was conjugated to one of the free secondary amine groups of spermine (1 eq. oleic acid, 1 eq. DCC, and 1 eq. DMAP). Purification of the mono-acylated spermine was followed by flash chromatography. The second linoleoyl chain was added using DCC coupling of linoleic acid to the 1,12-diphthalimido-*N*^9^-oleoyl-4,9-diazadodecane (1 eq. linoleic acid, 1 eq. DCC, and 1 eq. DMAP). Deprotection of the phthalimide protecting groups then followed by refluxing in hydrazine monohydrate in DCM/THF 1:1 mixture to obtain *N*^4^-linoleoyl*-N*^9^-oleoylspermine, which was purified by flash chromatography [23]. The guanidinylation of amines typically involves an electrophilic amidine group as part of the guanidinylating reagent [25]. 1,3-Di-Boc-2-(trifluoromethylsulfonyl)guanidine was used as it can carry out the guanidinylation of primary and secondary amines under mild conditions [25,26]. The guanidinylation was carried out on the di-acylated spermine derivatives and deprotection of the Boc protected guanidine group was carried out using TFA to obtain the trifluoroacetate salts of the synthesized compounds (Figure 3).

### 3.2. Lipoplex particle size and ζ-potential

The lipoplexes prepared at the cationic lipid/siRNA ratios for each guanidinylated lipid which resulted in the best reduction in GFP expression were chosen to be characterized for their particle size and ζ-potential (Table 1). Particle size measurement using dynamic light scattering showed that the particle size varied from 132–575 nm. The two cationic lipids **3** and **4** which are acylated with unsaturated C18 fatty acids (dioleoyl and linoleoyl/oleoyl respectively) and which showed the best reduction in GFP expression, had particle sizes of 303 and 158 nm respectively. The particle size of the C22 (dierucoyl) conjugate **1** was the smallest (132 nm) while the short chain C12 (dilauroyl) conjugate **2** had the largest particle size of 575 nm. Lipoplex size has been identified as an important factor in transfection efficiency, although not the only determinant factor [27]. Lipoplexes within size range 200–300 nm have been previously reported [28]. Although the size of the lipoplexes will determine the main route of entry with smaller lipoplexes (<300 nm) likely to enter via clathrin mediated endocytosis, and larger particles (>500 nm) entering cells via caveoli mediated endocytosis [28,29], one recent report shows that the actual entry route for functional siRNA mediated gene silencing might possibly be fusion with the plasma membrane rather than the endocytosis pathway [30]. The ζ-potentials measurements showed that all the lipoplexes had positive values within the range 28-50 mV. Cationic lipids **3** and **4** had the similar ζ-potential of 45 mV. Positive ζ-potential is important in promoting stability of the prepared lipoplexes by enhancing repulsion between the nanoparticles. Although having positive ζ-potential will promote interaction between the positively charged lipoplexes and the negatively charged groups present on the cell membrane surface, it was reported that in the presence of serum, the lipoplexes actually acquire a negative ζ-potential [28] while still maintaining efficient transfection efficiency.

### 3.3. siRNA binding (RiboGreen intercalation assay)

An siRNA binding assay was used to evaluate the ability of the synthesized guanidinylated lipids **1**, **2**, **3**, and **4** to complex and bind siRNA. The assay depends on the increased fluorescence (approx. 1000-fold) of bound RiboGreen dye compared to the free (unbound) dye which is practically non-fluorescent [31]. The loss of fluorescence compared to control siRNA indicates the binding of siRNA to cationic lipids and hence prevention of RiboGreen binding with siRNA which leads to reduction of fluorescence compared to the control (free) siRNA [32,33]. The four cationic lipids **1-4** efficiently bound siRNA and the normalised fluorescence, relative to free siRNA (100%), was reduced to: 5 ± 2 (**1**), 12 ± 3 (**2**), 0 ± 1 (**3**), and 8 ± 2 (**4**). These results prove that the guanidinylated lipids are able to efficiently bind siRNA.

### 3.4. Transfection with siRNA and evaluating delivery and knock-down

HeLa cells that was previously transfected to stably express GFP was used to evaluate the siRNA delivery and sequence specific knock-down of GFP expression. The siRNA against GFP used was labelled with Alexa Fluor 647 (AF647) in the 3′-position of the anti-sense strand to enable simultaneous tracking of the siRNA delivery and reduction of GFP expression by measuring the fluorescence of the AF647 and the GFP during the FACS analysis. A healthy population of sample cells were gated before recording the fluorescence during FACS.

**Figure 4.**
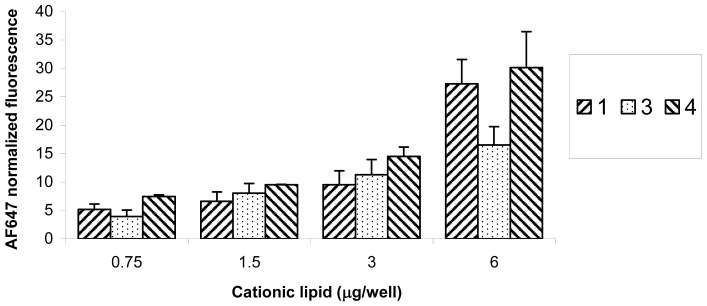
Delivery of siRNA (15 nM, 15 pmol/well) against GFP (labelled with Alexa Fluor® 647 at the 3′-position of the sense strand) using **1**, **3**, and **4**. Values are presented as means of normalized geometric mean fluorescence of AF647 ± SD (n = 6).

The normalized fluorescence of AF647 measured 48 h post transfection was measured as an estimate for the delivered amount of siRNA. Figure 4 shows that, for each cationic lipid, there is a general trend of increasing fluorescence by increasing the amount of the lipid. Cationic lipid **2** data are not shown due to the very low siRNA delivery. With respect to **1**, **3**, and **4**, there was a significant statistical difference between the geometric mean AF647 fluorescence measured at 3 and 6 μg/well (N/P = 10 and 20 respectively) with p < 0.05. There was also a significant statistical difference between the amounts of siRNA delivered (geometric mean fluorescence of AF647) by **3** and **4** (p < 0.05) with lipoplexes formulated at 6 μg/well (N/P = 20). There was no significant statistical difference between **1** and **4** at 6 μg/well (p = 0.33). These results show that, given that **1**, **3**, and **4** have two guanidine head-groups in the form of trifluoroacetate salts in common, the C18 (18:1 and 18:2) unsaturated fatty acids conjugated at positions *N*^4^ and *N*^9^ of the parent spermine provided the optimum chain length for siRNA delivery compared to the C12 (12:0) and C22 (22:1) chains.

**Figure 5.**
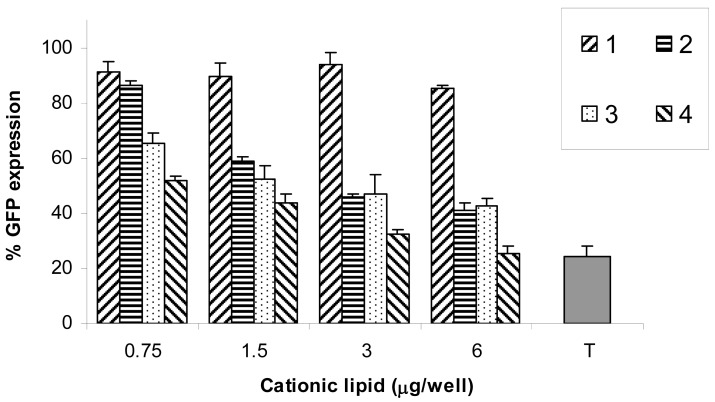
Reduction in GFP expression in HeLa cells after transfection with lipoplexes of **1**, **2**, **3**, and **4** at different cationic lipid/siRNA ratios. siRNA concentration is kept constant (15 nM, 15 pmol/well). Values are presented as mean ± SD (n = 6). Commercial TransIT TKO (T) is shown for comparison.

Figure 5 shows that the best reduction of GFP expression was achieved by **4** followed by **3**. At 6 μg/well, GFP expression was reduced to 26% and 43% for **4** and **3** respectively (p < 0.05). Lipid **1** did not show any practically significant reduction in GFP (reduced to 85%) at 6 μg/well (N/P = 18). Lipid **2** resulted in GFP reduction to 46% at 6 μg/well (N/P = 26), however, this reduction cannot be evaluated without considering the high toxicity of **2** which will affect the expression of GFP, as will be discussed later. Lipids **3** and **4** with C18 (18:1 and 18:2) resulted in the best knock-down of GFP expression, which might be attributed to the fusogenic ability of unsaturated fatty acids (in *cis*-configuration) which favours (L_α_ to H_II_) transition as they can promote both membrane fusion and endosomal escape [23,34,35]. The chain length is an important factor that affects the efficiency of GFP knock-down because although the C22 (22:1) has one centre of unsaturation, lipid **1** resulted in less reduction in GFP (85%) compared to **3** (43%) and **4** (26%) with significant statistical difference between the compared means (p < 0.05) at 6 μg/well. The chain length affect siRNA delivery and siRNA mediated knock-down in a different manner, as evident from comparing the delivery of **1**, **3**, and **4** and their GFP reduction at a concentration of 6 μg/well. Although **1** and **4** resulted in similar siRNA delivery efficiencies, lipid **4** was much better than **1** in terms of GFP reduction (to 26% and 85% respectively). These differences in gene silencing efficiency compared with cellular uptake of the lipoplexes may reflect the multi-step processes of gene silencing and/or more than one mechanism of cell entry [30].

Also, lipid **3** resulted in better GFP reduction when compared to **1** (to 43% and 85% respectively) despite the fact that **1** resulted in higher siRNA delivery compared to **3** (p < 0.05). The importance of chain length and chain unsaturation of cationic lipids has been previously reported to be among the most significant factors that affect transfection efficiency because of the effect on the hydrophobic volume of lipid and its hydrophilic/lipophilic ratio which will in turn affect the properties of the formed lipoplexes [36].

When compared to the commercial transfecting agent TransIT TKO, the reduction in GFP expression obtained with lipoplexes of **4** (26%) was the same as that obtained with TransIT TKO (24%), *i.e.*, there was no significant statistical difference (p = 0.30).

**Figure 6.**
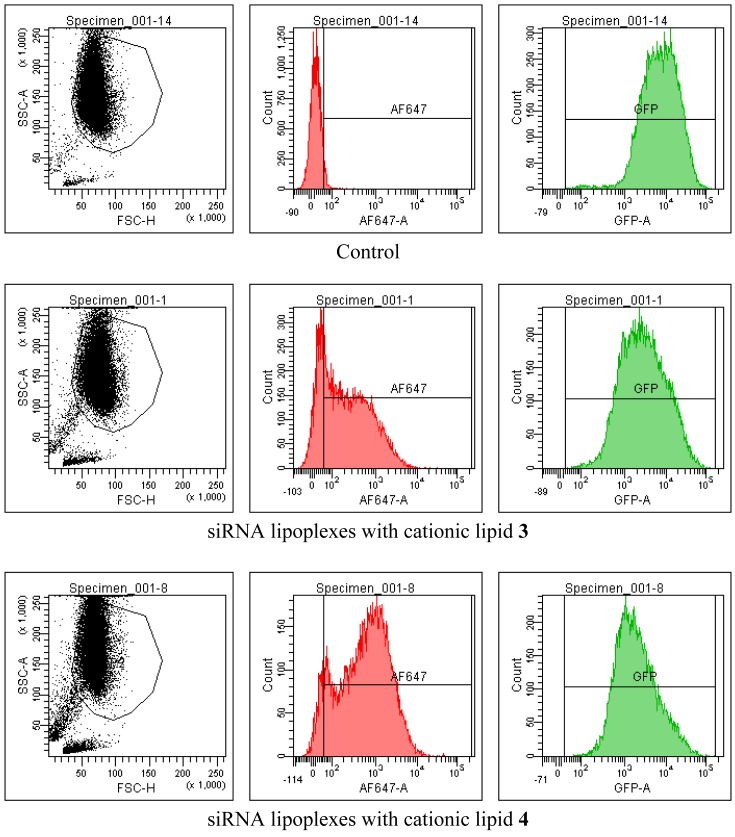
Gated FACS analysis of delivery of siRNA (15 nM, 15 pmol/well) against GFP (labelled with Alexa Fluor® 647 at the 3′-position of the sense strand) and GFP expression in HeLa cells 48 h post transfection with lipoplexes of **3** and **4** (6 μg/well) at an siRNA concentration of 15 pmol/well. The AF647 gate (red) shows 75% of parent-gated cells with **3**, and 92% of parent-gated cells with **4**. The GFP gate (green) shows silencing to ∼40% and ∼25% respectively measured by geometric mean fluorescence relative to control (top line).

The increases in fluorescence shown in Figure 4 reflect increases in siRNA delivery with increasing concentrations of cationic lipids in the lipoplexes up to an N/P ratio of 20 (6 μg/well). Figure 5 shows the corresponding reduction in GFP expression. These data were obtained from gated FACS analyses of the healthy populations of HeLa cells (parent gate), representative examples of which are shown in Figure 6 together with the percentage of cells transfected. In order to evaluate siRNA delivery, the AF647 gate was set-up to include cells that have fluorescence signals higher than the auto-fluorescence of control cells detected at λ = 660 nm. The GFP gate was set to calculate the geometric mean fluorescence of GFP.

**Figure 7.**
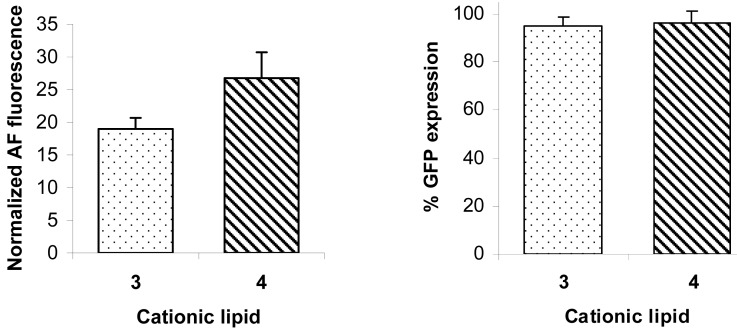
Scrambled AllStars siRNA (with an Alexa Fluor® 647 tag at the 3′-position) (15 nM, 15 pmol/well) was delivered with cationic lipids **3** and **4** (at 6 μg/well). Delivery of tagged siRNA (left) is expressed as AF647 normalized geometric mean fluorescence ± SD (n = 6) measured at λ = 660 nm. GFP percentage expression (right) (absence of silencing as a negative control) 48 h post transfection is measured at λ = 530 nm.

The effects of transfecting HeLa cells using **3** and **4** with scrambled siRNA on lipoplex delivery and GFP expression are shown in Figure 7. Qiagen report that their scrambled siRNA lacks any homology to mammalian genes. Figure 7 shows that the GFP expression was practically not affected by the transfection process while the scrambled siRNA was delivered in comparable amounts (*i.e.*, comparable normalized fluorescence) to delivery of the siRNA against GFP. Thus, cationic lipids **3** and **4** deliver two different siRNAs with similar efficiency. These results prove that GFP reduction after transfection with siRNA against GFP (shown as averaged data in Figure 5 and as representative examples in Figure 6) is due to sequence specific gene silencing and not due to any toxic effects of the cationic lipid vectors.

### 3.5. Confocal microscopy cell imaging

**Figure 8.**
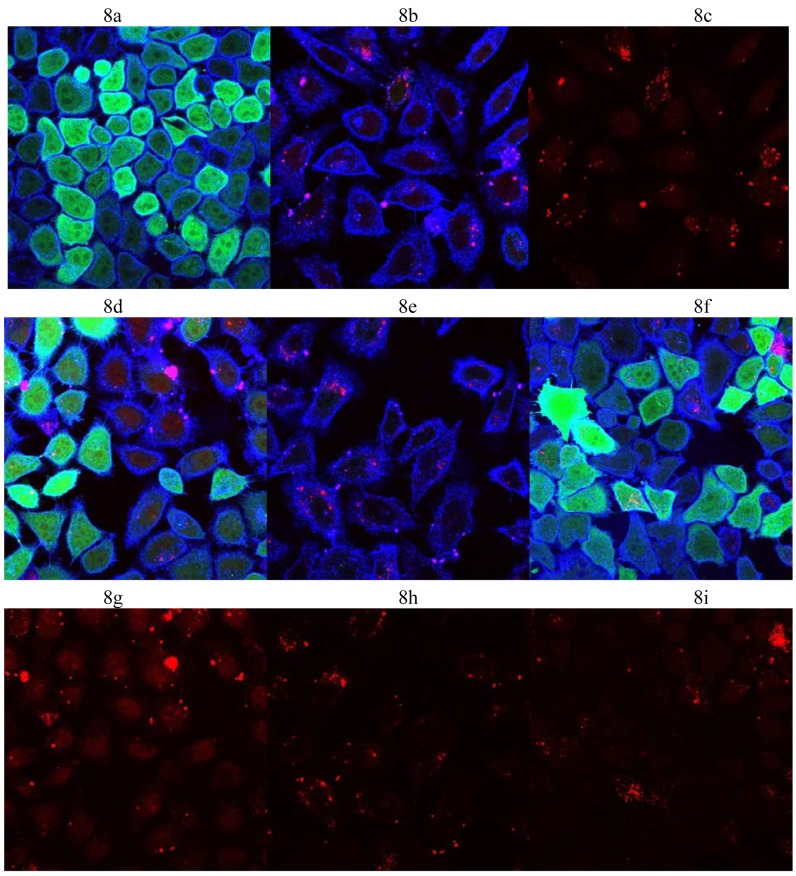
Confocal microscopy cell imaging. GFP fluorescence (green), cell membrane stained with WGA-Alexa Fluor® 555 (blue), and Alexa Fluor® 647 (red) represents tagged siRNA delivery. (**a**) non-transfected HeLa cells (control); (**b**) reduction in GFP expression after transfection with siRNA against GFP delivered with **3** (6 μg/well); (**c**) as b, but only the red channel; (**d**): as b, but using scrambled siRNA; (**e**) reduction in GFP expression after transfection with siRNA against GFP delivered with **4** (6 μg/well); (**f**) as e, but using scrambled siRNA; (**g**): as d, but only the red channel; (**h**) as e, but only the red channel; (**i**) as f, but only the red channel.

Figure 8 shows confocal microscope images of HeLa cells after transfection with **3** and **4** using siRNA against GFP or scrambled siRNA at 6 μg/well of cationic lipid which is the amount of lipid that resulted in the best reduction of GFP expression with respect to each of the cationic lipids.

Figure 8a shows control HeLa cells. Figure 8b shows the reduction of GFP expression after transfection with siRNA against GFP using **3** at 6 μg/well. Figure 8c is the same as 8b, but only the red channel is turned on to track better the delivery of the AF647 labelled siRNA against GFP. It can be seen that the AF647 fluorescence (red) is distributed throughout the cell and concentrated in some cell areas. Figure 8d shows that lipoplexes of **3** did not cause reduction in GFP expression when using scrambled siRNA which was delivered to HeLa cells successfully as shown in Figure 8g. These results prove that the siRNA was delivered successfully to the HeLa cells and that the reduction in GFP expression is due to sequence specific knock-down of GFP and not due to any toxic effects of the cationic lipid vectors. The same conclusion can be obtained when examining the transfection of HeLa cells with **4** as shown in Figure 8e with 8h, and 8f with 8i.

### 3.6. Cell viability assay

**Figure 9.**
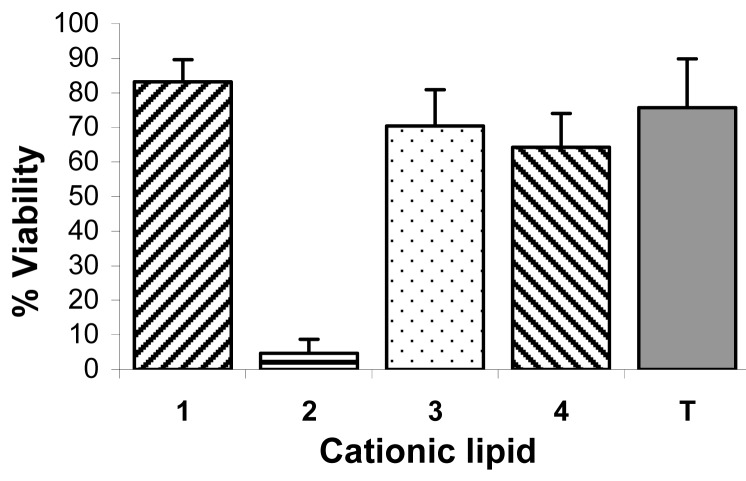
Comparison of cell viability of HeLa cells after transfection with lipoplexes at their optimal cationic lipid/siRNA ratios and compared with the commercially available transfection agent TransIT TKO (T). Values are presented as mean ± SD (n = 6). Experiments were carried out at: 0.6 μg/well synthesized cationic lipids, 1.5 pmol siRNA/well (15 nM), and 6,500 cells/well.

Following transfection with cells seeded at a density of 65,000 cells/well in 24-well plates, *i.e.* 65,000 cells/1 mL, cell viability was assayed in 96-well plates with 6,500 cells/0.1 mL [23,37]. The ratio of cells to the amount of cationic lipids used, the concentration of cationic lipids (0.6 μg/0.1 mL) and of siRNA (15 nM) were exactly as used in the transfection experiments [23,38]. Figure 9 shows that **1**, **3**, and **4** resulted in more than 64% cell viability. There were no statistical significant difference between the cell viability of **3** and **4** (p = 0.32) with cell viabilities of 70% and 64% respectively. The best cell viability, obtained by **1** (83%), was significantly different (p < 0.05) from the cell viability of **3** and **4**. Whilst diacylated C12 (12:0) **2** is a new compound, the very high toxicity of its parent diamine, *N*^4^, *N*^9^-dilauroylspermine, has been previously reported in both HtTA cells [39] and HeLa cells [23] with scrambled siRNA. There were no significant differences between cell viability of TransIT TKO (76%) and **3** (64%), p = 0.12, or between TransIT TKO and **4** (70%), p = 0.41. There is a probability that the counter ion, trifluoroacetate in this case, has a contributing negative effect on cell viability as been reported before [40] where the presence of residual TFA in the concentration range 10^−8^ to 10^−7^ M resulted in reduction of cell proliferation of osteoblasts, chondrocytes, and neonatal mice calvariae.

## 4. Conclusions

The four synthesized diguanidinylated diacylated spermine-based cationic lipids were able to bind siRNA efficiently and to form particles with sizes in the nanometre range (132–575 nm). Saturated shorter chain (C12:0) **2** showed relatively high toxicity when compared with the longer chain (C18-C22) *N*^1^,*N*^12^-diamidino-*N*^4^,*N*^9^-diacylated spermine derivatives. Transfection with self-assembled siRNA lipoplexes of **3** and **4** resulted in the sequence specific knock-down of GFP in HeLa cells, exhibiting comparable (low) toxicity to the commercial transfecting agent TransIT TKO. Lipid **4** with one linoleoyl and one oleoyl chain, acylated on positions *N^4^* and *N^9^* respectively of the *N*^1^,*N*^12^-diamidinospermine, was the best transfecting agent. Lipoplexes of lipid **4** showed the same efficiency, in HeLa cells, in terms of reduction of GFP expression as TransIT TKO.

In this article, we have described the synthesis of four novel spermine-derived fatty acid amide guanidines applied to the self-assembly of siRNA lipoplexes which were then tested in GFP expressing HeLa cells. The major conclusions include detection of siRNA complexion in the lipoplexes, cellular uptake, toxicity, and gene silencing efficiency even in the presence of serum. This is a structure-activity relationship (SAR) study in siRNA delivery of which there are few reported; a recent contribution being the design, synthesis, and analysis of spermine-siRNA conjugates containing two oleylamine carbamate chains [41].

## Figures and Tables

**Table 1.** *N*^1^,*N*^12^-Diamidine derivatives of different lipospermines. The fatty acids are described by two numbers separated by a colon, first the chain length and then the number of double bonds. Particle size and **ζ-**potential of guanidinylated lipospermines were measured at the cationic lipid/short interfering RNA (siRNA) ratios that showed best reduction in GFP expression.

**Name of compound**	**Fatty acid**	**Description of fatty acid**	**Particle size (nm) ± SD**	**Zeta-potential (mV) ± SD**
*N^1^*,*N^12^*-Diamidino*-N*^4^,*N*^9^-dierucoylspermine **1**	Erucic	22:1	132 ± 4	50 ± 1
*N^1^*,*N^12^*-Diamidino-*N*^4^,*N*^9^-dilauroylspermine **2**	Lauric	12:0	575 ± 61	28 ± 3
*N^1^*,*N^12^*-Diamidino-*N*^4^,*N*^9^-dioleoylspermine **3**	Oleic	18:1	303 ± 6	45 ± 3
*N^1^*,*N^12^*-Diamidino-*N*^4^-linoleoyl*-N*^9^-oleoylspermine **4**	Linoleic and Oleic	18:2 and 18:1	158 ± 24	45 ± 3
